# First evidence of plastic fallout from the North Pacific Garbage Patch

**DOI:** 10.1038/s41598-020-64465-8

**Published:** 2020-05-06

**Authors:** Matthias Egger, Fatimah Sulu-Gambari, Laurent Lebreton

**Affiliations:** The Ocean Cleanup Foundation, Rotterdam, The Netherlands

**Keywords:** Environmental sciences, Environmental monitoring, Pollution remediation, Ocean sciences, Marine chemistry

## Abstract

The infamous garbage patches on the surface of subtropical oceanic gyres are proof that plastic is polluting the ocean on an unprecedented scale. The fate of floating plastic debris ‘trapped’ in these gyres, however, remains largely unknown. Here, we provide the first evidence for the vertical transfer of plastic debris from the North Pacific Garbage Patch (NPGP) into the underlying deep sea. The numerical and mass concentrations of plastic fragments (500 µm to 5 cm in size) suspended in the water column below the NPGP follow a power law decline with water depth, reaching values <0.001 pieces/m^3^ and <0.1 µg/m^3^ in the deep sea. The plastic particles in the NPGP water column are mostly in the size range of particles that are apparently missing from the ocean surface and the polymer composition of plastic in the NPGP water column is similar to that of floating debris circulating in its surface waters (i.e. dominated by polyethylene and polypropylene). Our results further reveal a positive correlation between the amount of plastic debris at the sea surface and the depth-integrated concentrations of plastic fragments in the water column. We therefore conclude that the presence of plastics in the water column below the NPGP is the result of ‘fallout’ of small plastic fragments from its surface waters.

## Introduction

Plastic debris can harm marine life through a multitude of pathways, from releasing of toxic chemicals to animal entanglement, choking and starving of wildlife after ingestion, and distributing of non-native and potentially harmful organisms^1^. Ocean plastic pollution is therefore a major environmental problem, creating an urgent need for an understanding of the transport and transformation of plastic debris in marine systems, to improve risk assessments and inform possible mitigation solutions. As of 2017, the global annual plastic production exceeded 348 million metric tons^2^, or ~400 million metric tons if synthetic polymers used in spinning textile fibers are included^3^. Growing worldwide plastic consumption results in a rapid buildup of plastic waste in the environment. In a business-as-usual scenario, the amount of annually generated mismanaged plastic waste could triple by mid-century, reaching 155–265 million metric tons^4^. Each year, a fraction of this waste, that is a few million metric tons, eventually enters the sea from coastal environments^5^ and rivers^6,7^. From the onset of mass production of synthetic polymers in the 1950s, total emissions of positively buoyant plastics into the marine environment have amounted to tens of million metric tons^5,3^. Yet, measurement-based estimates of buoyant plastic debris currently afloat at sea range in the order of hundreds of thousands of metric tons^8–10^. In essence, these quantities of floating plastic account for less than 1% of all the plastic ever estimated to have entered the ocean. One of the most pressing questions in ocean plastic pollution research therefore concerns the whereabouts of the 99% ‘missing plastic’^11,12^.

To date, the largest mass concentrations of positively buoyant plastic waste have been reported for the surface ocean in subtropical latitudes. Transported by currents, wind and waves, floating plastic objects eventually accumulate at the sea surface of remote subtropical oceanic gyres^8–10^. With peak mass concentrations of hundreds of kilograms per km^2^ and highest numerical plastic concentrations exceeding one million pieces per km^2^ ^9,10,13–16^ for particles >500 µm in size, the surface waters of the subtropical gyres are often referred to as ocean ‘garbage patches’. The presence of decades-old objects found in the North Pacific gyre^13^ indicates that floating plastic pollution in these waters may be highly persistent. However, a better understanding of the plastic inputs and outputs is crucial to more accurately assess the residence time and the fate of positively buoyant plastics accumulating in these regions. Current estimates of plastic input from land to the ocean are based on reported country-scale statistics on municipal waste generation, and assume a range of conversion rates from waste into marine inputs of plastic^5^. The dynamics of release into the marine environment, however, remain poorly understood. Importantly, not all positively buoyant plastic objects reach offshore waters^17^. Coastal environments play a critical role in pre-gyre removal of floating plastic debris. Beaching onto coastlines removes part of the floating debris from the sea surface relatively quickly after these objects have entered the ocean^17–19^. Results from a recent whole-ocean emission-transport-degradation model suggest that a large part (>66%) of the plastic mass released from land into the ocean since the 1950s could have stranded or settled around the world’s shoreline, possibly slowly circulating between coastal environments in repeated episodes of beaching, fouling, de-fouling and resurfacing^19^. The stranding, settling and resurfacing in the coastal ocean is hypothesized to lead to pre-gyre natural selection of debris, where only a fraction of debris with certain characteristics such as polyethylene (PE) or polypropylene (PP) plastic objects with low surface to volume ratio and low windage coefficients accumulates in subtropical gyres^13^.

Plastic objects at sea fragment into smaller micro-sized particles by the action of the sun, waves, temperature variations and marine organisms^20^. The observed size distribution of floating plastic debris collected at the sea surface shows that submillimeter-sized fragments are present in smaller concentrations than anticipated from the fragmentation of larger debris^9^. This apparent loss of small microplastics suggests that there are size-selective sink mechanisms at play removing floating microplastic debris from the surface waters^8^. A possible sink mechanism for microplastics is the colonization by organisms (i.e. biofouling), which can reduce the buoyancy of small floating plastic fragments, characterized by higher surface to volume ratio, eventually resulting in a positive settling velocity for initially buoyant particles^21,22^. In deeper water layers, the debris can subsequently undergo de-fouling, allowing the plastic to repeatedly sink and then resurface as floating debris^23,24^. Buoyant microplastics can also be ingested by marine life^25,26^, incorporated into marine snow^27^ and fecal pellets^28^, as well as form aggregations with marine biogenic particles^29,30^ and suspended inorganic particles^31^. These interactions can result in the sedimentation of small floating plastic fragments away from the ocean surface. However, the amplitude of these interactions is largely unknown and subsurface mass reservoirs remain unconstrained. As such, the long-term fate of plastic debris accumulating in the subtropical gyres essentially remains a mystery.

**Figure 1:**
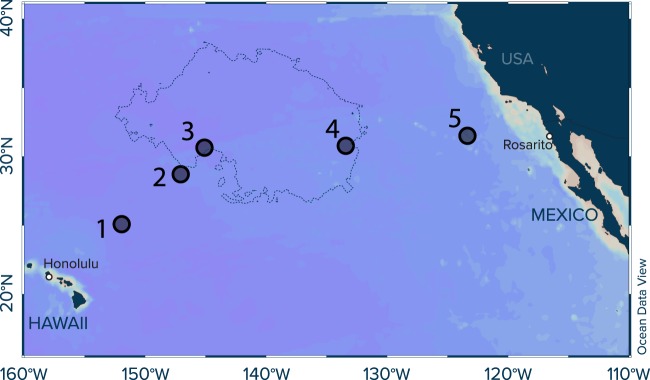
Study sites located in the eastern North Pacific Ocean. Concurrent surface and underwater trawling (down to 2000 m water depths) was performed at five stations along a cruise transect from Honolulu (Hawaii, USA) to Rosarito (Mexico) to study the vertical distribution of plastic debris (500 µm to 5 cm in size). The predicted boundaries of the North Pacific Garbage Patch (defined as microplastic concentrations >1 kg/km^2^) are shown as dashed lines and taken from^13^.

In this study, we provide the first water column concentration profiles (0–2000 m water depth) of plastic particles between 500 µm and 5 cm in size in the subtropical eastern North Pacific Ocean. Samples were collected at five stations along a cruise transect from Honolulu (USA) to Rosarito (Mexico) (Fig. 1, Supplementary Table S1), traversing the North Pacific Garbage Patch (NPGP). Our results suggest that the plastic particles found in the water column are likely fragments originating from the fallout of initially buoyant plastic debris circulating in the surface waters of the NPGP. We thus report an observational evidence of the whereabouts of the missing smaller fraction of plastic debris floating at the surface of subtropical oceanic gyres.

## Methods

### Sampling

Vertical concentration profiles of plastic debris (>500 µm) were collected at five locations onboard the Maersk Transporter during the deployment and monitoring phase of The Ocean Cleanup’s System 001 (“Wilson”) in November-December 2018 (Fig. 1). The five stations were selected to represent a gradient in floating plastic concentrations along a cruise transect from Honolulu (Hawaii, USA) to Rosarito (Mexico) based on predicted spatial patterns of plastic pollution for the region^9,10,13^. Important metadata and a detailed sampling scheme per site are shown in Supplementary Table S1. At each Station, a CTD (Conductivity, Temperature, Depth) profiler (Sea-Bird Electronics, SBE-5T) was lowered to 2000 m water depth using the stern A-frame, measuring temperature, salinity, dissolved oxygen and fluorescence. The water column profiles of these parameters are provided in Supplementary Fig. S1 and were used to identify distinct water layers of interest such as the mixed layer, the chlorophyll maximum, the North Pacific Intermediate Water layer characterized by a well-defined salinity minimum at depths of 300–1000 m^32^, the oxygen minimum zone, and the deep-sea. After identification of the targeted water layers, underwater net tows at different water depths were performed from the stern A-frame using a Multiple Opening and Closing Net with an Environmental Sensing System (MOCNESS) consisting of a total of 9 individual 333 µm square mesh size nets (Biological Environmental Sampling Systems, Inc.). The net width was 1 m and the net mouth area at a 45° towing angle equaled 1 m^2^. First, the MOCNESS was lowered to 2000 m (1500 m at Station 1 and 2) with the first net open (i.e. net #0). Note that any samples towed in net #0 were not used for further analyses due to possible contamination with plastic fragments from the surface waters. Once the MOCNESS reached the deepest depth, net #0 was closed and net #1 opened. The MOCNESS was then slowly hauled towards the sea surface and eight specific water layers were sampled (nets #1 to #8). The trawled depth trajectories are shown in Supplementary Fig. S2 and the corresponding coordinates and filtered water volumes are presented in Supplementary Table S1. The vessel speed was adjusted to maintain a 45° towing angle of the MOCNESS at all times, and typically fluctuated around 2 knots. An in-build mechanical flow meter recorded the filtered water volume of each individual net tow. Back on deck, the content of the individual MOCNESS cod-ends (333 µm mesh size) was immediately transferred into single-use cod-ends (333 µm mesh size). The latter were sealed with staples, placed in individual zip-lock bags, wrapped in aluminum foil and stored frozen until further analysis in the onshore laboratory.

Concurrent to the MOCNESS underwater trawling, a Manta trawl (Ocean Instruments, Inc.) was deployed from the starboard crane to collect data on plastic concentrations in the surface waters. The Manta trawl mouth area was 90 cm ×15 cm (width x height) and the net mesh size was 500 µm (square mesh). Three subsequent trawls, each 30 min in duration, were performed and sampled water volumes were recorded using a mechanical flow meter (General Oceanics, Inc.). Note that at Station 1, the Manta trawl was only deployed once. After each deployment, the net was rinsed from the outside with freshwater and the single-use cod-end (333 µm mesh size) removed, sealed with staples, placed in individual zip-lock bags, wrapped in aluminum foil and stored frozen (−20 °C) until further analysis in the onshore laboratory.

### Sample processing

All trawled samples were analyzed according to the procedure defined in^13^ to enable comparability with previous sampling efforts in the North Pacific Ocean. The content of each cod-end was washed into a sieve tower, separating the material into the following size classes: 500 µm – 1.5 mm (small microplastics), 1.5 – 5 mm (large microplastics), 5 mm – 1.5 cm (small mesoplastics), 1.5 – 5 cm (large mesoplastics), and >5 cm. Note that objects >5 cm are excluded from the subsequent analyses due to a sampling bias towards smaller objects associated with neuston trawls^13^. The sieves were then placed separately into round aluminum tins filled with filtered (<1 µm) seawater from the North Atlantic Ocean (salinity 35) and the sieve content was stirred manually until all debris particles were detached from the biomass material. Subsequently, floating objects identified as buoyant anthropogenic debris according to the criteria described in^33^ were hand-picked using stainless steel tweezers and their widest dimension was measured with a ruler to account for thin but long debris that may have slipped through the square sieve apertures. If larger in size than the sieve fraction, the object was placed into the appropriate size class corresponding to the widest dimension of the object. Each object was further classified and tallied into one of the following type categories: (1) ‘H-type’ for fragments and objects made of hard plastic, plastic sheet or film; (2) ‘N-type’ for fragments of plastic lines, ropes, and fishing nets; (3) ‘P-type’ for pre-production plastic pellets in the shape of a cylinder, disk or sphere; and (4) ‘F-type’ for fragments or objects made of foamed material (e.g. expanded polystyrene). Once counted and categorized, the objects were washed with water purified by reverse osmosis, transferred to aluminum dishes, dried at 60 °C for 3 hours, and weighed using an OHAUS Explorer EX324M scale (0.0001 g readability). Subsequently, the dried debris items were analyzed individually by Raman spectroscopy (Agiltron, Inc.; PeakSeeker PEK-785) to identify the corresponding plastic polymer type. If the number of particles per size class and type category exceeded 10 pieces, a random subset of 10 particles was analyzed. In total, 305 particles were analyzed by Raman spectroscopy and the sample spectra subsequently compared to an in-house Raman plastic reference library. The latter was built by analyzing plastic samples from household items with a known polymer composition on the same Raman spectroscope, covering the main polymer types accumulating in the marine environment (i.e. PP, HDPE, LDPE, PS, PVC, PET, cellulose acetate and nylon). Furthermore, spectra were collected for paint samples from the vessel and trawling equipment, as well as for the MOCNESS and Manta trawl nylon nets in order to rule out possible contamination with particles from these materials. For 26 particles, no conclusive Raman spectra were obtained, mostly because these particles were black in color resulting in strong absorption of the Raman laser and subsequent burning of the particle surface. These 26 particles were further analyzed by Fourier Transform Infrared Spectroscopy (Thermo Scientific; Nicolet 6700 FTIR) at Utrecht University.

### Contamination control

Contamination of samples with microplastics during sampling and laboratory analyses is a major challenge in microplastic research. The risk of contamination is especially high when working with microplastic particles <500 µm in size and with synthetic fibers. In this study, we focus on microplastic particles >500 µm only. Furthermore, we did not include any microfibers in our analyses, as these fibers typically pass through the trawling nets deployed here. Thus, possible contamination with airborne fibers (a major source of microplastic contamination) does not impact our results. Consequently, the work was performed outside a glove box or laminar flow cabinet. To minimize contamination with plastic fragments during sampling and laboratory analyses, standard non-plastic laboratory and fieldwork equipment such as metal and glass were used, and the samples always remained covered with clean aluminum foil when not in use. Furthermore, all nets (Manta trawl and each individual MOCNESS net) and all cod-ends were thoroughly rinsed from the outside with freshwater prior to each deployment and carefully inspected for the presence of (micro)plastic particles. Although running procedural blanks is not possible when collecting samples by trawling, we can rule out contamination by fibers broken off the nylon nets as we did not observe any white (Manta trawl) or black (MOCNESS) nylon fibers in the cod-end samples. We did, however, observe some plaques of color (light blue, red and black). These paint particles were likely scratched off the vessel wall (light blue and red) and MOCNESS frame (black) during deployment of the equipment. The paint particles were clearly distinguished from plastic fragments based on their Raman (light blue and red) and FTIR (black) spectra, and thus not included in the subsequent analyses. Lastly, the seawater used in the laboratory was filtered through a sequence of filters (<20 µm, <5 µm, <1 µm) and all laboratory equipment (sieves, tweezers, aluminum tins and dishes) was thoroughly rinsed and carefully inspected for cleanliness prior to each use.

### Calculation of plastic concentrations

The numerical and mass concentrations of plastic items (count/kg of plastic per volume of water/sea surface area) measured by each net tow were calculated for each plastic size and type category separately. The towed area was calculated by multiplying the net mouth area (0.135 m^2^ and 1 m^2^ for the Manta trawl and MOCNESS, respectively) by the tow length (determined from the flowmeter and from vessel GPS position data). The average tow length (±1 standard deviation) for the Manta net tows was 2.065 ± 0.443 km, while the average tow length for each MOCNESS net tow was 2.249 ± 0.636 km.

Positively buoyant plastic items are mixed within the upper water column due to wind-induced turbulent mixing^34,35^. As a result, plastic items collected by Manta trawling may underestimate the total amount of buoyant plastics in the area sampled, especially at higher sea states. Kukulka *et al*. (2012)^34^ developed a one-dimensional model that predicts the vertical distribution of buoyant plastic particles at different sea states. Their model can be applied to calculate depth-integrated numerical and mass concentrations for the Manta trawl measurements, thus accounting for wind-driven mixing of buoyant plastics at the sea surface using the following equation^34^:

1 $${C}_{i}=\frac{{C}_{s}}{1-{e}^{-d{W}_{b}{\left(1.5,\sqrt{\frac{{\rho }_{a}}{{\rho }_{w}}{C}_{d}{U}^{2}},k,\frac{0.96}{g},{\sigma }^{\frac{3}{2}},{C}_{d},{U}^{2}\right)}^{-1}}}$$

Where *C*_*i*_ represents the depth-integrated concentration for the upper 5 m of water column (in pieces/kg per volume of water/sea surface area), *C*_*s*_ represents the concentration of a plastic type and size category as measured by the Manta trawl (in pieces/kg per volume of water/sea surface area), *d* is the depth sampled by the Manta trawl (equal to 0.15 m), ${W}_{b}$ is the terminal rising velocity of plastic within a plastic type and size category (in m/s) taken from^13^, ${\rho }_{a}$ is the air density (in kg/m^3^), ${\rho }_{w}$ is the seawater density (in kg/m^3^), ${C}_{d}$ is the drag coefficient (equal to 0.0012), $U$ is the wind speed during sampling (in m/s), $k$ is the Karman constant (equal to 0.4), $g$ is the gravitational constant (equal to 9.81 m/s^2^), and $\sigma$ is the wave age, equal to 35 (assuming a fully developed sea state). Values for depth-integrated concentrations were estimated using average wind speeds equal to 0, 2, 5, 9, 13 and 19 knots for sampling events associated with Beaufort sea states 0, 1, 2, 3, 4, and 5, respectively, and the median values for rising velocity measurements provided in^13^. For a detailed description of the applied correction for wind-induced mixing of positively buoyant plastics, the reader is referred to^13^.

### Power law function for water column concentration profiles

**Figure 2:**
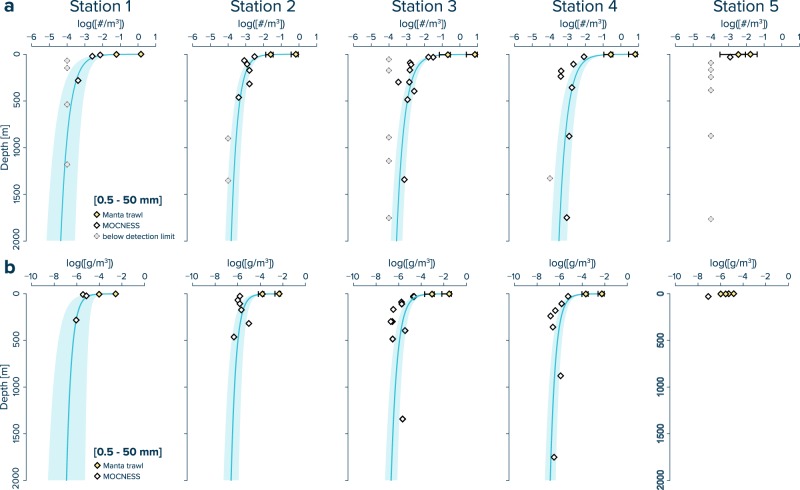
Water column profiles of (**a**) numerical and (**b**) mass concentrations of plastic particles (500 µm to 5 cm in size). Data collected with Manta trawls (yellow diamonds) are presented for the upper 0.15 m (net opening) of the ocean surface, and as values corrected for wind-induced mixing in the upper 5 m of water column, with average concentrations and whisker extending to the smallest and largest concentrations measured. Note that only one Manta trawl was deployed at Station 1. Gray diamonds represent MOCNESS underwater trawls in which no plastic fragments were found (detection limit of ~10^–4^ #/m^3^; equal to one particle per filtered water volume). The blue lines and shaded areas represent the power law functions and associated 95% confidence intervals derived from log-log correlations between water depth and plastic concentrations shown in Fig. 3. Note that at Station 5, concentrations of water column plastic particles were too low to derive a reliable vertical distribution function.

We developed a simple vertical model to reproduce the observed water column profiles of plastic debris at each station. The model is based on the measured depth dependence between plastic concentrations and water depth (Fig. 2). In this approach, the plastic concentrations (C) are calculated as a function of water depth according to:

2 $$C=\,{10}^{(a\times \log (depth)+b)}\times CF$$

**Figure 3:**
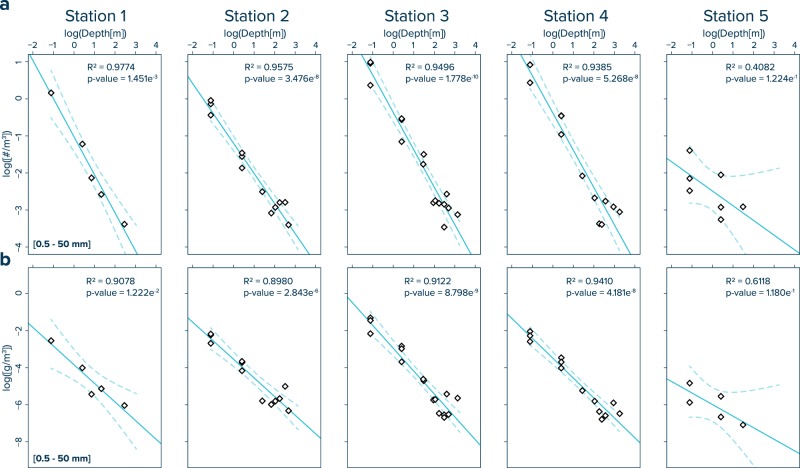
Empirical relationships used to model water column plastic concentrations. Log-log plots of water depth vs. (**a**) numerical and (**b**) mass concentrations of plastic fragments (>500 µm). The blue solid line represents the linear regression fit and the dashed lines indicate the 95% confidence interval of the regression. The regression equations are presented in Supplementary Table S2.

Where $a$ and $b$ are the slope and intercept of the regression line derived from the log-log plot between observed plastic concentrations (mass and numerical) and the corresponding water depth (Fig. 3). A correction factor (CF) is applied to account for the skewness bias inherent in the back conversion from a log-log transformed linear regression model to arithmetic units. As described comprehensibly by Middelburg and colleagues^36^, high values lose significance relative to lower values in a log-log linear regression model. Applying a log-log linear regression to arithmetic units as done here thus results in a skewness bias towards lower values. The CF corrects for this bias and can be calculated using the variance of the model residuals^36^: CF = e^(2.65*variance)^. The parameters $a$, $b$, and CF for each station are shown in Supplementary Table S2. The correction factors vary between 1.16 and 1.62 for the numerical concentration models and between 1.70 and 2.41 for the mass concentration models (Supplementary Table S2). Such values are within the range of correction factors previously derived for empirical biogeochemical models in marine sediments^36^.

## Results

### Ocean surface

A total of 11,879 items (size range: 500 µm to 5 cm) were collected at the ocean surface by Manta trawls. Observed numerical concentrations of floating plastic debris increased from a few hundreds of thousands of items (#) per km^2^ at Stations 1 (300,768 #/km^2^) and 2 (126,574 ± 53,663 #/km^2^) to more than one million items per km^2^ at Stations 3 (1,062,493 ± 618,808 #/km^2^) and 4 (1,345,687 ± 692,833 #/km^2^), before decreasing again to 17,699 ± 23,177 items per km^2^ at Station 5 (Fig. 2 and Supplementary Table S3). The corresponding mass concentrations were 0.490 kg/km^2^, 0.797 ± 0.400 kg/km^2^, 4.571 ± 3.229 kg/km^2^, 1.070 ± 0.625 kg/km^2^ and 0.005 ± 0.008 kg/km^2^ for Stations 1 to 5, respectively (Fig. 2 and Supplementary Table S3). These figures are consistent with previous sampling efforts in the region^13,14,16^. Additionally, we find high spatial variability in the surface concentrations, with numerical concentrations varying by up to one order of magnitude between two subsequent Manta trawl deployments (Supplementary Table S3). Such highly variable plastic concentrations on an ocean sub mesoscale have also been reported in earlier studies^14,16^, highlighting the heterogeneity of plastic accumulation at the sea surface.

For all stations, fragments and objects made of hard plastic (‘H-type’) were the dominant debris type, followed by fragments of plastic lines, ropes and fishing nets (‘N-type’). Pre-production plastic pellets (‘P-type’) were exclusively found in the 0.15 – 0.5 cm size fraction, and no expanded polystyrene fragments (‘F-type’) were found (Supplementary Table S3). The polymer composition of ocean plastic collected with the Manta trawls was dominated by polyethylene (PE) and polypropylene (PP), accounting for 92% (PE) and 8% (PP) of the analyzed samples at Station 1, 96% (PE) and 4% (PP) at Station 2, 94% (PE) and 6% (PP) at Station 3, 91% (PE) and 9% (PP) at Station 4, and for 100% (PE) at Station 5, respectively (Table 1). The dominance of PE and PP is in line with previous observations on plastic pollution in the NPGP^13^.

**Table 1:** Polymer composition of plastic particles collected in this study.

	# fragments	PE	PP	PVC	PS	unknown
Manta 1	53	92%	8%	—	—	—
Manta 2	27	96%	4%	—	—	—
Manta 3	31	94%	6%	—	—	—
Manta 4	32	91%	9%	—	—	—
Manta 5	7	100%	—	—	—	—
MOCNESS 1	3	33%	33%	—	—	33%
MOCNESS 2	20	65%	25%	5%	5%	—
MOCNESS 3	90	83%	10%	—	1%	6%
MOCNESS 4	39	97%	—	—	3%	—
MOCNESS 5	3	67%	—	—	33%	—

PE – polyethylene, PP – polypropylene, PVC - polyvinylchloride, PS – polystyrene.

### Ocean water column

Concentrations of plastic particles in the water column exceeded the detection limit of ~10^–4^ particles per m^3^ at all sites. In total, 184 plastic fragments (size range: 500 µm to 5 cm) were collected by multinet underwater trawling (MOCNESS - Multiple Opening and Closing Net with an Environmental Sensing System), where 3 particles were counted at Station 1, 22 particles at Station 2, 117 particles at Station 3 (note that the multinet was deployed twice at Station 3; see Supplementary Table S1), 39 particles at Station 4 and 3 particles at Station 5. The fragments were all classified as H-type (68%) and N-type (32%) objects (Supplementary Table S4). Most of the H-type fragments (i.e. 76.2%) were between 0.05 – 0.15 cm in size, with the remaining 21.4% and 2.4% of H-type particles attributed to the 0.15 – 0.5 cm and 0.5 – 1.5 cm size classes, respectively. The size class distribution for the N-type fragments showed a larger variability, with 29.3% of fragments in the 0.15 – 0.5 cm, 53.4% in the 0.5 – 1.5 cm and 17.2% in the 1.5 – 5 cm size range, respectively. Note, however, that the absence of N-type fragments in the smallest size class (0.05 – 0.15 cm) is likely due to loss during trawling as these thin fibers can easily slip through the cod-end mesh. Also note that the longest dimension is reported here.

Similar to the plastic fragments collected with the Manta trawls at the sea surface, the polymer composition of plastic debris in the ocean water column was dominated by PE and PP, accounting for 87% and 10% of the identified particles, respectively. The remaining particles were identified as polyvinylchloride (1%) and polystyrene (3%). For six particles, no conclusive spectra were obtained, likely because of a high degree of weathering.

Our results further reveal a positive correlation between the plastic concentrations in the surface water and the deepest depth where plastic debris was found. Plastic particles (>500 µm) were present down to water depths of 281 m at Station 1 (trawled depth interval: 189–436 m), 462 m at Station 2 (trawled depth interval: 397–599 m), 1340 m at Station 3 (trawled depth interval: 1192–1494 m), 1749 m at Station 4 (trawled depth interval: 1496–2002 m), and 30 m at Station 5 (trawled depth interval: 3–64 m), respectively. Numerical concentrations decreased exponentially with water depth, from 0.060 #/m^3^ (Station 1), 0.025 ± 0.011 #/m^3^ (Station 2), 0.212 ± 0.124 #/m^3^ (Station 3), 0.269 ± 0.139 #/m^3^ (Station 4), and 0.004 ± 0.005 #/m^3^ (Station 5) in the upper 5 m of water column, to below 0.001 #/m^3^ in the deep sea (Fig. 2, Supplementary Tables S3 and S4). Mass concentrations follow a similar power law decline with water depth, decreasing from 98 µg/m^3^ (Station 1), 159 ± 80 µg/m^3^ (Station 2), 914 ± 646 µg/m^3^ (Station 3), 214 ± 125 µg/m^3^ (Station 4), and 1 ± 2 µg/m^3^ (Station 6) in the surface waters to around ~ 0.1 µg/m^3^ at depth in the water column (Fig. 2, Supplementary Tables S3 and S5).

The numerical and mass concentrations of collected plastic fragments show log-log linear correlations with the corresponding water depth, with R^2^ values ranging from 0.8980 to 0.9774 for Stations 1–4 (Fig. 3). At Station 5, the correlation between water depth and plastic concentrations is associated with larger uncertainty due to the low concentrations of plastic debris at the sea surface and the absence of detectable plastic fragments (detection limit ~10^–4^ particles per m^3^) in the deeper water column. Consequently, Station 5 is excluded from subsequent vertical modelling. The regression model parameters and associated power law function equations are presented in Supplementary Table S2. Water column profiles calculated using these power law functions are shown as blue lines in Fig. 2. In general, the modelled numerical and mass plastic concentration are in good agreement with the measured water column distribution, with 100% (numerical) and 94% (mass) of the modelled values deviating less than one order of magnitude from the observations (Supplementary Fig. S3).

**Figure 4:**
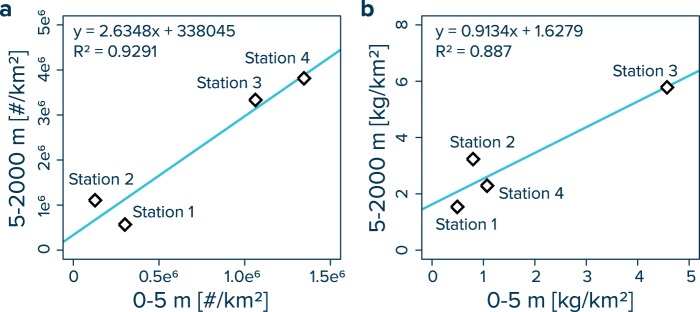
Areal depth integrated concentrations for plastic debris in the 0–5 m and 5–2000 m water masses. Values on the x-axes represent (**a**) numerical and (**b**) mass concentrations of floating plastic debris (500 µm to 5 cm in size) at the ocean surface collected by Manta trawls and corrected for wind-induced mixing in the upper 5 m of water column. Values on the y-axes are the depth-integrated concentrations for the water column below (5–2000 m water depth) as derived by integrating the corresponding power law functions (Fig. 2; Supplementary Table S2) using integration steps of 1 m. The values are provided in Supplementary Table S6. Note that no power law function is available for Station 5.

Integrating the power law functions for the vertical distribution of plastic particles enables the conversion of volumetric water column concentrations (i.e. #/m^3^ and kg/m^3^) to areal depth-integrated concentrations (i.e. #/km^2^ and kg/km^2^), thus permitting the direct comparison of plastic quantities between different water masses. Using 1 m vertical integration steps, we calculate average concentrations of 0.57 million plastic particles per km^2^ for the water column between 5–2000 m at Station 1, 1.11 million plastic particles per km^2^ for Station 2, 3.33 million plastic particles per km^2^ for Station 3 and 3.81 million plastic particles per km^2^ for Station 4, respectively (Supplementary Table S6). The corresponding mass concentrations for the 5–2000 m water layer are 1.537 kg/km^2^, 3.232 kg/km^2^, 5.782 kg/km^2^ and 2.288 kg/km^2^ for Stations 1 to 4, respectively (Supplementary Table S6). Plotting the integrated concentrations of the 5–2000 m water layer against the corresponding numerical and mass concentrations in the surface ocean (i.e. 0–5 m) reveals that the quantity and mass of plastic fragments in the ocean water column increases with increasing concentrations of floating plastic debris at the sea surface (Fig. 4).

Comparing the observed vertical distribution of plastic fragments with measured water column profiles of salinity, temperature, dissolved oxygen and fluorescence did not reveal any significant correlations between these parameters (Supplementary Fig. S4). Interestingly, however, our data could indicate somewhat elevated concentrations of plastic at water depths of around ~300–400 m (i.e. in the upper North Pacific Intermediate Water layer at Stations 2, 3 and 4).

## Discussion

To date, studies on plastic pollution in the eastern North Pacific Ocean have mainly focused on positively buoyant plastic debris afloat in the surface ocean^8,9,13,14,16^. This study dives deeper into the water column and provides the first vertical view on the extent of ocean plastic pollution in the NPGP, a major plastic accumulation zone in the subtropical waters between California and Hawaii^14^. Our results reveal that concentrations of plastic fragments between 500 µm and 5 cm in size show a power law decline with water depth, with numerical concentrations up to four and mass concentrations up to five orders-of-magnitude higher at the ocean surface compared to deeper water layers (Figs. 2 and 3). Most (i.e. 76%) of the plastic particles found in the water column were smaller than 5 mm in size, with 52% of the particles smaller than 1.5 mm. Intriguingly, the water column particles were dominated by PE and PP (Table 1). Earlier observations of the size distribution of floating plastic debris collected at the ocean surface reported that millimeter- and submillimeter-sized fragments are underrepresented when compared to expected degradation rates from larger debris^8,9^, suggesting a loss of small microplastics from the sea surface. Thus, we find that plastic particles in the NPGP water column are mostly in the size range of particles that are apparently missing from the ocean surface and the polymer composition of plastic in the NPGP water column is similar to that of floating debris circulating in its surface waters. These results suggest that plastic particles found below the NPGP are likely fragments originating from initially buoyant plastic debris accumulating in the NPGP. We further observe a positive correlation between the amount of plastic debris at the sea surface and the depth-integrated concentrations of plastic fragments in the water column (Fig. 4). Taken together, we can conclude that the presence of plastic debris in the water column below the NPGP is the result of ‘fallout’ of initially positively buoyant microplastics from the surface waters.

**Figure 5:**
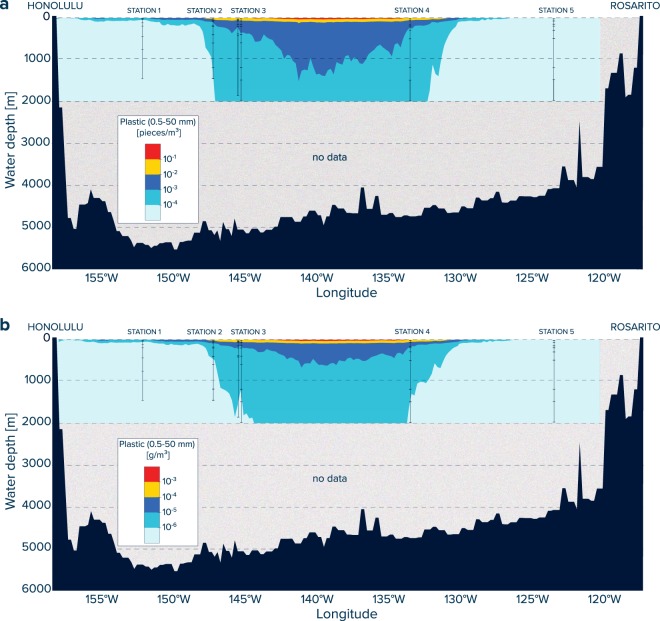
Predicted concentrations of plastic debris in the upper 2000 m of ocean water column along our cruise track from Honolulu (Hawaii, USA) to Rosarito (Mexico). The (**a**) numerical and (**b**) mass concentrations in the water column are estimated as a function of the debris afloat at the ocean surface in the eastern North Pacific Ocean^13^ and corresponding water depth (see Supplementary Fig. S5). The sampling stations and associated trawling depths are indicated by black solid lines. Note that these predictions do not consider possible temporal variations and are missing measurements in areas with highest concentrations of floating plastic debris. Thus, these plots should be interpreted as a first qualitative attempt to visualize the possible vertical distribution of plastic debris in the area.

The positive correlation between surface and water column-integrated concentrations indicates that the vertical structure of the plastic profiles may be approximated from the amount of plastic debris afloat at the ocean surface. For this, the modelled plastic concentrations (blue lines in Fig. 2) are normalized to the corresponding plastic concentrations at the sea surface (i.e. in the upper 5 m of water column). These concentration ratios can subsequently be used to establish an empirical relationship between surface and subsurface concentrations (Supplementary Fig. S5). We derive the vertical distribution of plastic debris along our cruise transect (Fig. 5) from recent model estimates of surface concentrations of plastic in the eastern North Pacific Ocean for debris in the size range of 500 µm to 5 cm^13^. The result is a first attempt to visualize the possible vertical distribution of plastic debris in the region, depicting a limited horizontal transfer of plastic fragments at intermediate water depths and vertical transport processes possibly dominating the distribution of subsurface plastic pollution in the region. Note, however, that large uncertainties remain with such interpolations, which represent at best a snapshot of the vertical distribution of plastic debris (>500 µm) during the days of sampling in late 2018 and therefore may not be representative of temporal variations.

Our results provide first clues on the vertical extent of plastic pollution in the NPGP and hint at the possible plastic mass in the NPGP water column. Microplastic (0.5 – 5 mm) and mesoplastic (0.5 – 5 cm) debris has been shown to account for an estimated ~20% of the total plastic mass loading in the NPGP, with the other 80% attributed to plastic objects >5 cm in size^13^. We find that the depth-integrated mass concentrations of micro- and mesoplastics for water depths between 5–2000 m correspond to ~56–80% of the estimated micro- and mesoplastic mass in the upper 0–2000 m of the water column at our study sites (Supplementary Table S6). The relative mass distribution observed at our study sites therefore indicates that the mass of plastic debris suspended at water depths between 5–2000 m could be around one-tenth of the total plastic mass in the upper 0–2000 m of the NPGP water column. However, albeit little is known about the water column distribution of plastic debris and associated vertical fluxes of initially buoyant microplastics, it is likely that they show significant spatio-temporal variations^37^. Thus, more observational data spanning large spatial scales and considering temporal aspects are essential to reliably quantify the amount of plastic debris present in the water column below the NPGP and, more generally, in the global ocean.

Based on the data presented here, it remains to be shown whether the plastic particles in the water column below the NPGP are neutrally drifting, sinking towards the seabed or transported away laterally at deeper water layers. Such information is urgently needed to evaluate whether plastic concentrations in the water column below oceanic gyres will increase in the future due to ongoing fragmentation of larger plastic objects currently accumulating at the sea surface and subsequent sedimentation of initially floating microplastics. Somewhat elevated concentrations of microplastics at water depth of ~300 – 400 m (Fig. 2 and Supplementary Fig. S4) could indicate a lateral transport of small plastic fragments associated with the North Pacific Intermediate Water layer, or plastic accumulation at intermediate water depth due to vertical particle oscillations associated with biofilm growth and remineralization^24^. We recommend further investigation with a higher vertical sampling resolution, particularly in the upper few hundreds of meters, to evaluate possible dynamics of plastic transport and accumulation at these water depths. In addition, more research is needed to resolve the underlying mechanisms explaining the presence of floating plastic particles at such water depths. The underwater trawling used here results in the clumping of collected material in the cod-ends, making it difficult to evaluate whether the observed aggregations between organic matter and plastic fragments were already present *in-situ* or if they formed during sampling. For future studies, we therefore recommend installing sediment traps at various water depths below offshore plastic accumulation zones to quantify the vertical flux of plastic debris towards the deep ocean and to explore the associated vertical transport mechanisms. We further advocate for more research on settling velocities of plastic particles of various shapes and polymer types under different environmental conditions.

In summary, the results presented here provide strong evidence that the presence of plastic debris in the water column below subtropical oceanic gyres is the result of ‘plastic fallout’ from debris afloat in these offshore waters. Importantly, this study provides the observational base to quantify the fallout of microplastic from the ocean surface in future modelling work. Such knowledge is needed to assess the long-term fate of microplastics at sea and, ultimately, the persistency of ocean garbage patches.

## Supplementary information

Supplementary Information.

## Data Availability

All data needed to evaluate the conclusions in the paper are present in the paper and/or the Supplementary Materials.

## References

[1] UNEP. *Marine plastic debris & microplastics - Global lessons and research to inspire action and guide policy change*. (2016).

[2] PlasticsEurope. *Plastics – the facts*. (2018).

[3] Geyer R, Jambeck J, Law K (2017). Production, use, and fate of all plastics ever made. Sci. Adv..

[4] Lebreton L, Andrady A (2019). Future scenarios of global plastic waste generation and disposal. Palgrave Commun..

[5] Jambeck JR (2015). Plastic waste inputs from land into the ocean. Science (80-.)..

[6] Lebreton LCM (2017). River plastic emissions to the world’s oceans. Nat. Commun..

[7] Schmidt C, Krauth T, Wagner S (2017). Export of plastic debris by rivers into the sea. Environ. Sci. Technol..

[8] Cózar A (2014). Plastic debris in the open ocean. Proc. Natl. Acad. Sci..

[9] Eriksen, M. *et al*. Plastic pollution in the world’s oceans: more than 5 trillion plastic pieces weighing over 250,000 tons afloat at sea. *PLoS One* **9**, e111913 (2014). 10.1371/journal.pone.0111913 PMC4262196 25494041

[10] van Sebille E (2015). A global inventory of small floating plastic debris. Environ. Res. Lett..

[11] Cressey D (2016). The plastic ocean. Nature.

[12] Thompson RC (2004). Lost at sea: where is all the plastic?. Science (80-.)..

[13] Lebreton L (2018). Evidence that the Great Pacific Garbage Patch is rapidly accumulating plastic. Sci. Rep..

[14] Law KL (2014). Distribution of surface plastic debris in the eastern pacific ocean from an 11-year data set. Environ. Sci. Technol..

[15] Law KL (2010). Plastic accumulation in the North Atlantic Suptropical Gyre. Science (80-.)..

[16] Goldstein, M. C., Titmus, A. J. & Ford, M. Scales of spatial heterogeneity of plastic marine debris in the northeast Pacific Ocean. *PLoS One ***8** (2013). 10.1371/journal.pone.0080020 PMC3835860 24278233

[17] Van Sebille, E.*et al*. The physical oceanography of the transport of floating marine debris. *Environ. Res. Lett*. 1–54 (2020).

[18] Olivelli, A., Hardesty, B. D. & Wilcox, C. Coastal margins and backshores represent a major sink for marine debris: insights from a continental-scale analysis. *Environ. Res. Lett*. 10.1088/1748-9326/ab7836 (2020).

[19] Lebreton, L., Egger, M. & Slat, B. A global mass budget for positively buoyant macroplastic debris in the ocean. *Sci. Rep*. **9**, 1–10 (2019). 10.1038/s41598-019-49413-5 PMC6742645 31515537

[20] Andrady AL (2011). Microplastics in the marine environment. Mar. Pollut. Bull..

[21] Fazey FMC, Ryan PG (2016). Debris size and buoyancy influence the dispersal distance of stranded litter. Mar. Pollut. Bull..

[22] Fazey FMC, Ryan PG (2016). Biofouling on buoyant marine plastics: An experimental study into the effect of size on surface longevity. Environ. Pollut..

[23] Ye S, Andrady AL (1991). Fouling of floating plastic debris under Biscayne Bay exposure conditions. Mar. Pollut. Bull..

[24] Kooi M, Van Nes EH, Scheffer M, Koelmans AA (2017). Ups and downs in the ocean: effects of biofouling on vertical transport of microplastics. Environ. Sci. Technol..

[25] Clark JR (2016). Marine microplastic debris: a targeted plan for understanding and quantifying interactions with marine life. Front. Ecol. Environ..

[26] Choy CA, Drazen JC (2013). Plastic for dinner? Observations of frequent debris ingestion by pelagic predatory fishes from the central North Pacific. Mar. Ecol. Prog. Ser..

[27] Porter A, Lyons BP, Galloway TS, Lewis C (2018). Role of marine snows in microplastic fate and bioavailability. Environ. Sci. Technol..

[28] Cole, M. *et al*. Microplastics alter the properties and sinking rates of zooplankton faecal pellets. *Environ. Sci. Technol.* **50**, 3239–3246 (2016). 10.1021/acs.est.5b05905 26905979

[29] Long M (2015). Interactions between microplastics and phytoplankton aggregates: Impact on their respective fates. Mar. Chem..

[30] Michels, J., Stippkugel, A., Lenz, M., Wirtz, K. & Engel, A. Rapid aggregation of biofilm-covered microplastics with marine biogenic particles. *Proc. R. Soc. B Biol. Sci*. **285** (2018). 10.1098/rspb.2018.1203 PMC6125906 30158309

[31] Besseling E, Quik JTK, Sun M, Koelmans AA (2017). Fate of nano- and microplastic in freshwater systems: A modeling study. Environ. Pollut..

[32] Hansell DA, Carlson CA, Suzuki Y (2002). Dissolved organic carbon export with North Pacific Intermediate Water formation. Global Biogeochem. Cycles.

[33] Hidalgo-Ruz V, Gutow L, Thompson RC, Thiel M (2012). Microplastics in the marine environment: A review of the methods used for identification and quantification. Environ. Sci. Technol..

[34] Kukulka T, Proskurowski G, Morét-Ferguson S, Meyer DW, Law KL (2012). The effect of wind mixing on the vertical distribution of buoyant plastic debris. Geophys. Res. Lett..

[35] Reisser J (2015). The vertical distribution of buoyant plastics at sea: an observational study in the North Atlantic Gyre. Biogeosciences.

[36] Middelburg, J. J., Soetart, K. & Herman, P. M. J. Empirical relationships for use in global diagenetic models. *Deep Sea. Res. I* **44**, 327–344 (1997).

[37] Mountford, A. S. & Maqueda, M. Eulerian modeling of the three ‐ dimensional distribution of seven popular microplastic types in the global ccean. *J. Geophys. Res. Ocean*. **124** (2019).

