# Supplementary material for: First evidence of plastic fallout from the North Pacific Garbage Patch
Source: Sci Rep. 2020 May 6;10:7495. doi: 10.1038/s41598-020-64465-8 (PMC7203237; doi:10.1038/s41598-020-64465-8)
Supplement: Supplementary file 1 — Supplementary Information. [file 41598_2020_64465_MOESM1_ESM.pdf]

SUPPLEMENTARY INFORMATION

First evidence of plastic fallout from the North Pacific Garbage Patch

Matthias Egger,[1]* Fatimah Sulu-Gambari[1], Laurent Lebreton[1]

[1]The Ocean Cleanup Foundation, Rotterdam, The Netherlands

* corresponding author: matthias.egger@theoceancleanup.com

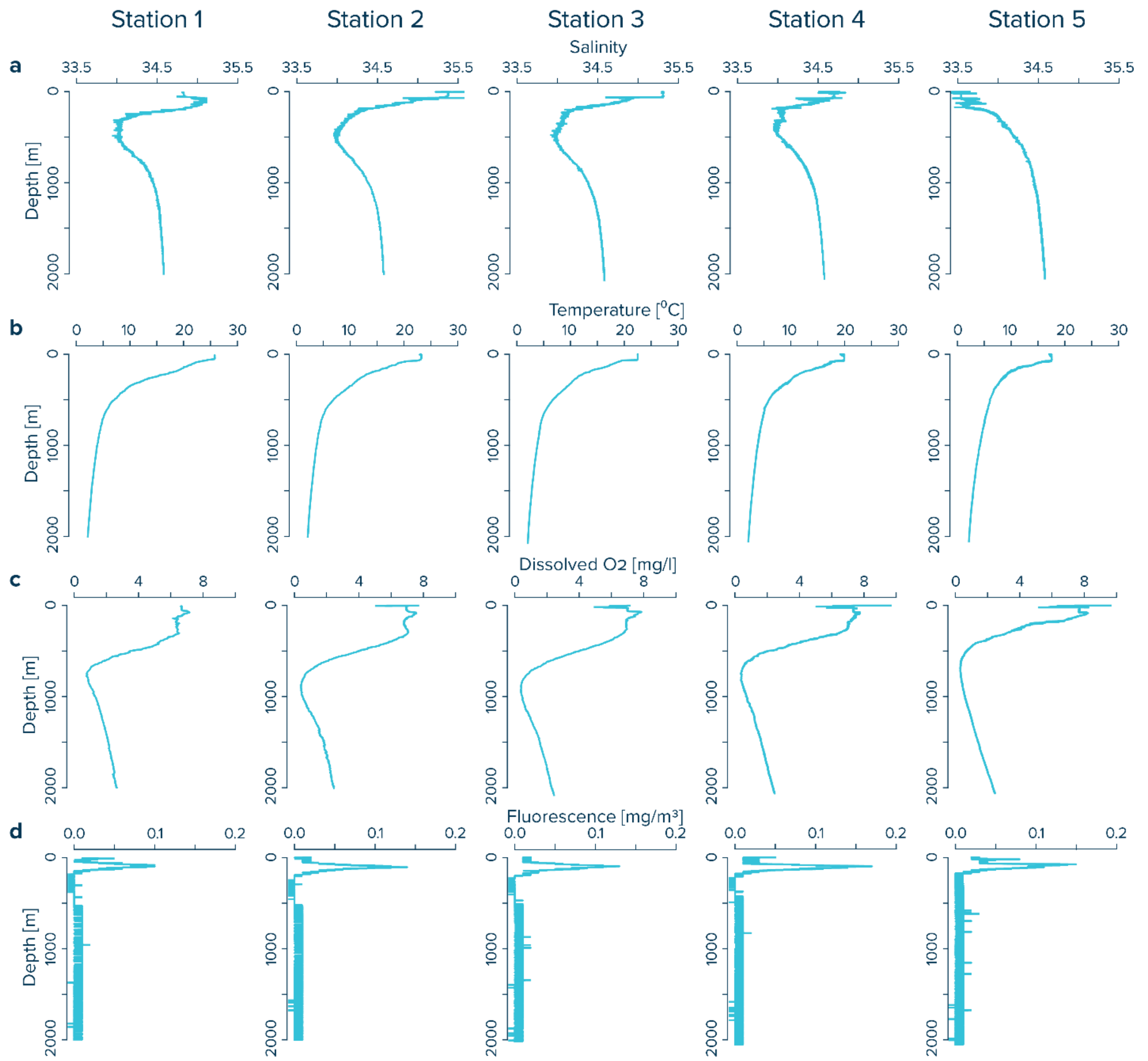

Fig. S1. Vertical CTD profiles. Water column profiles of (a) salinity, (b) temperature, (c) dissolved oxygen (O_2), and (d) fluorescence measured at each station prior to deployment of the MOCNESS. Note that the salinity minimum at depths of around 500 m indicates the North Pacific Intermediate Water layer [1].

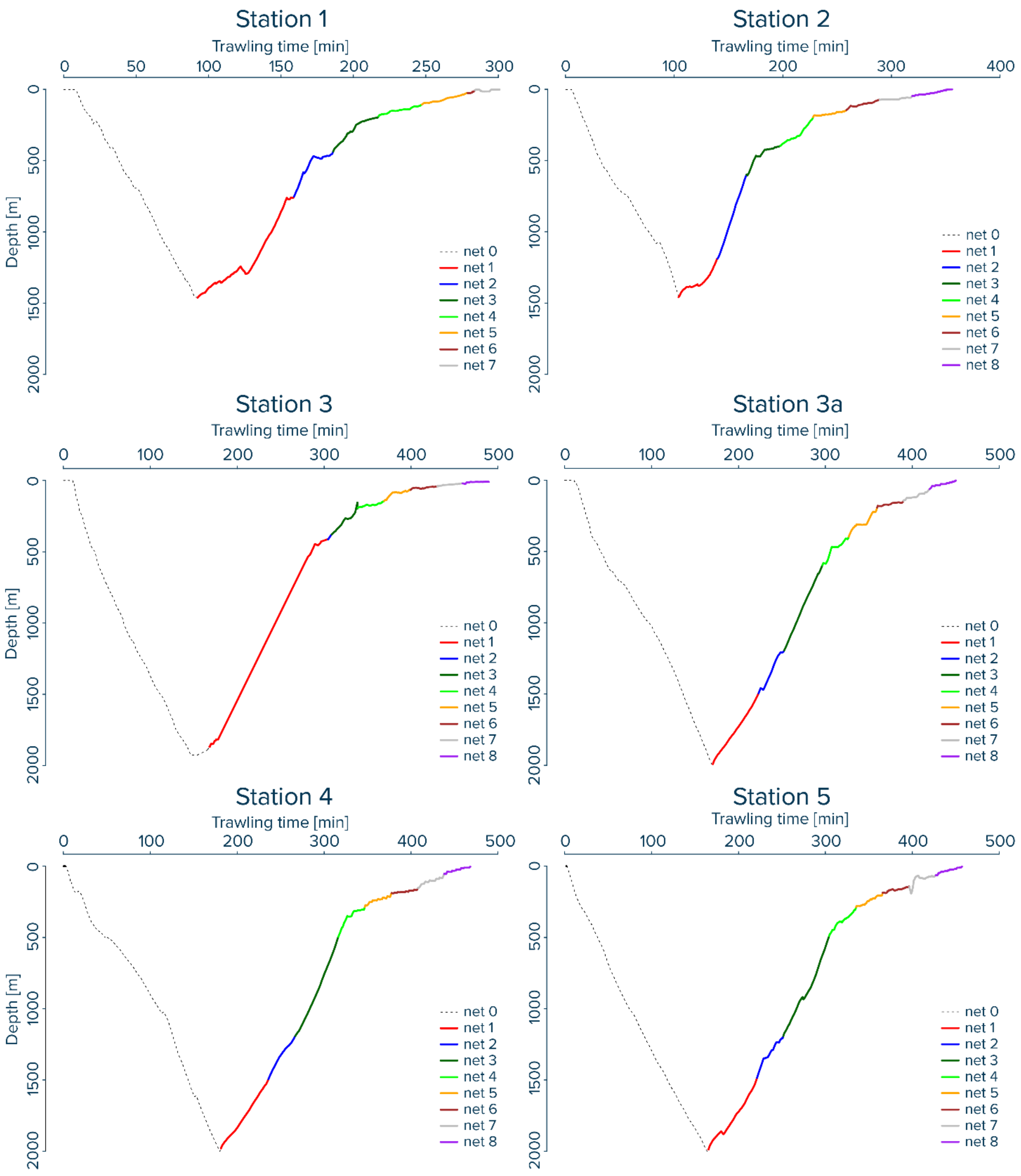

Fig. S2. MOCNESS trawling profiles. The MOCNESS applied in this study consisted of a total of 9 nets. To maintain a 45° towing angle, one net always needs to be open during trawling. The first sample (i.e. net #0) collected during descend of the MOCNESS was not included in the subsequent sample analyses due to possible contamination with plastic fragments from the sea surface. Sample volumes and coordinates of the individual net tows are provided in Supplementary Table S1.

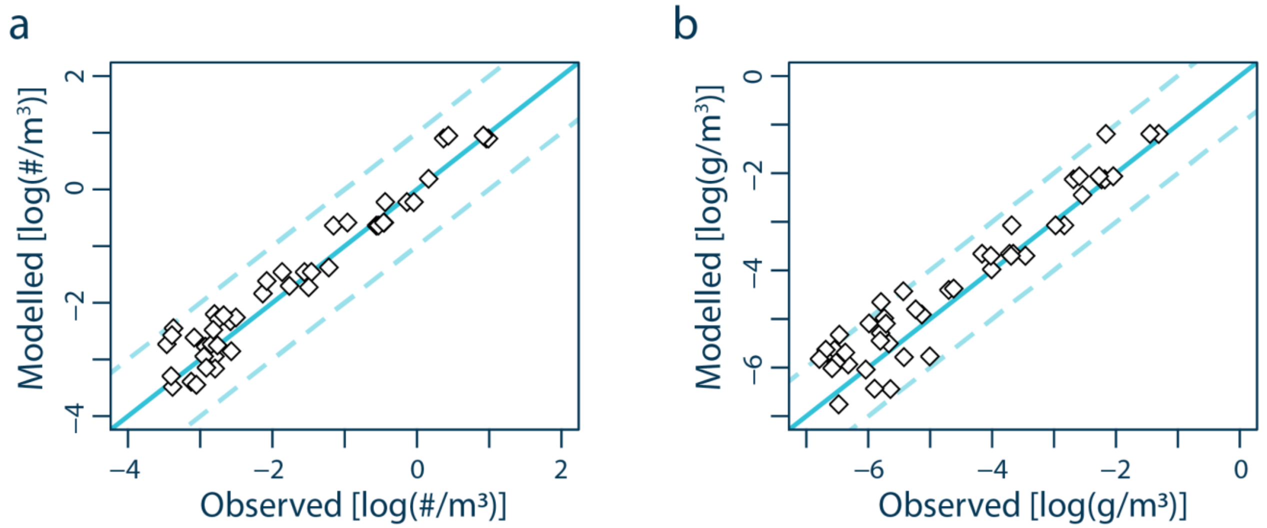

Fig. S3. Vertical model validation. Observed vs. modelled (a) numerical and (b) mass plastic concentrations. The blue solid line represents the 1:1 line (i.e. a perfect model fit) and the dashed lines indicates ± one order of magnitude deviation from the 1:1 line. The modelled concentrations are derived from applying the power law functions described in Supplementary Table S2.

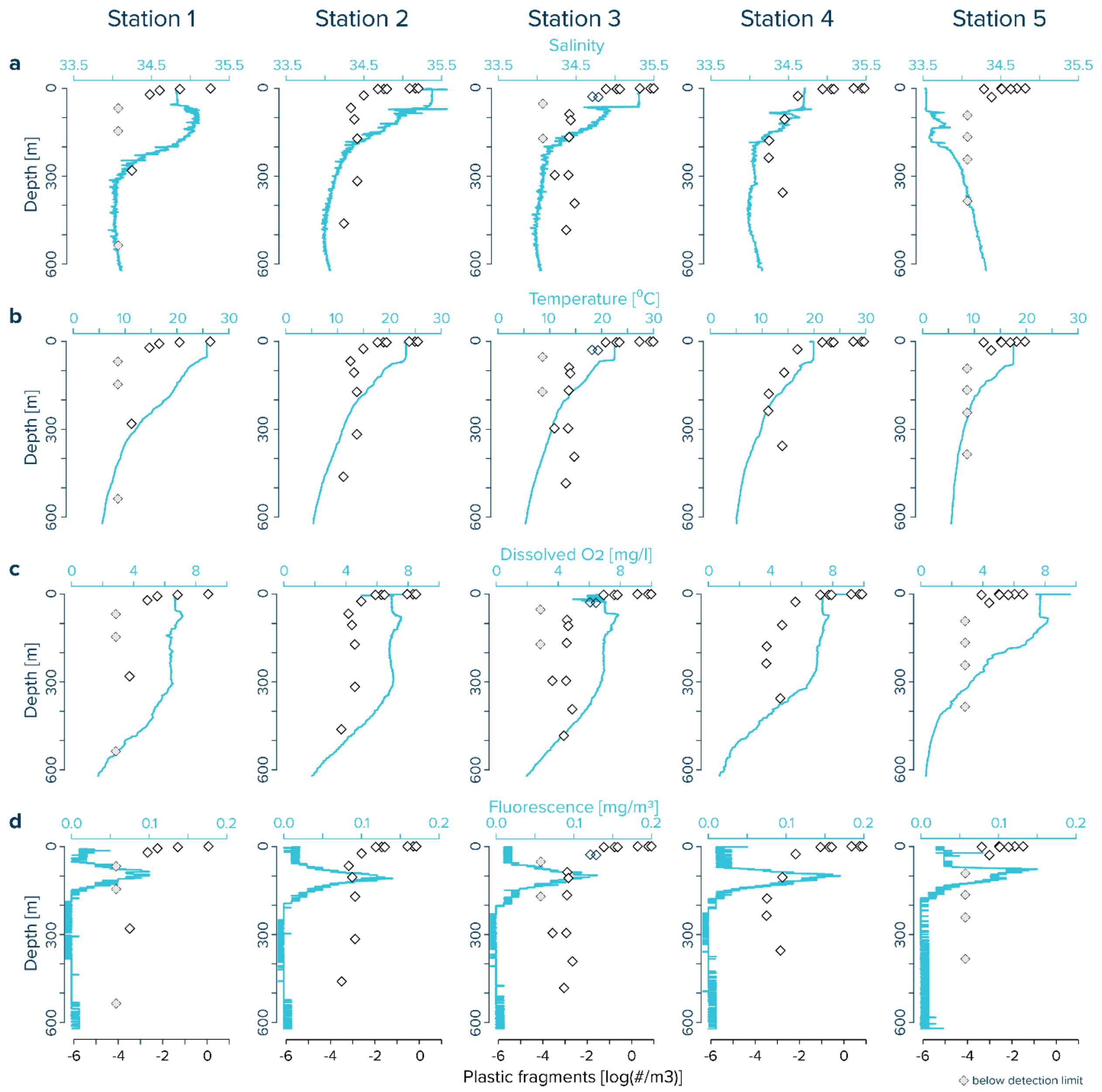

Fig. S4. Vertical CTD profiles and measured numerical concentrations of plastic fragments (500 µm to 5 cm in size) for the upper 600 m of water column. Water column profiles of (a) salinity, (b) temperature, (c) dissolved oxygen (O_2), and (d) fluorescence are presented as blue lines, while numerical plastic concentrations are shown as white diamonds. Gray diamonds represent MOCNESS underwater trawls in which no plastic fragments were found (detection limit of ~10^{-4} $\#/m^3$).

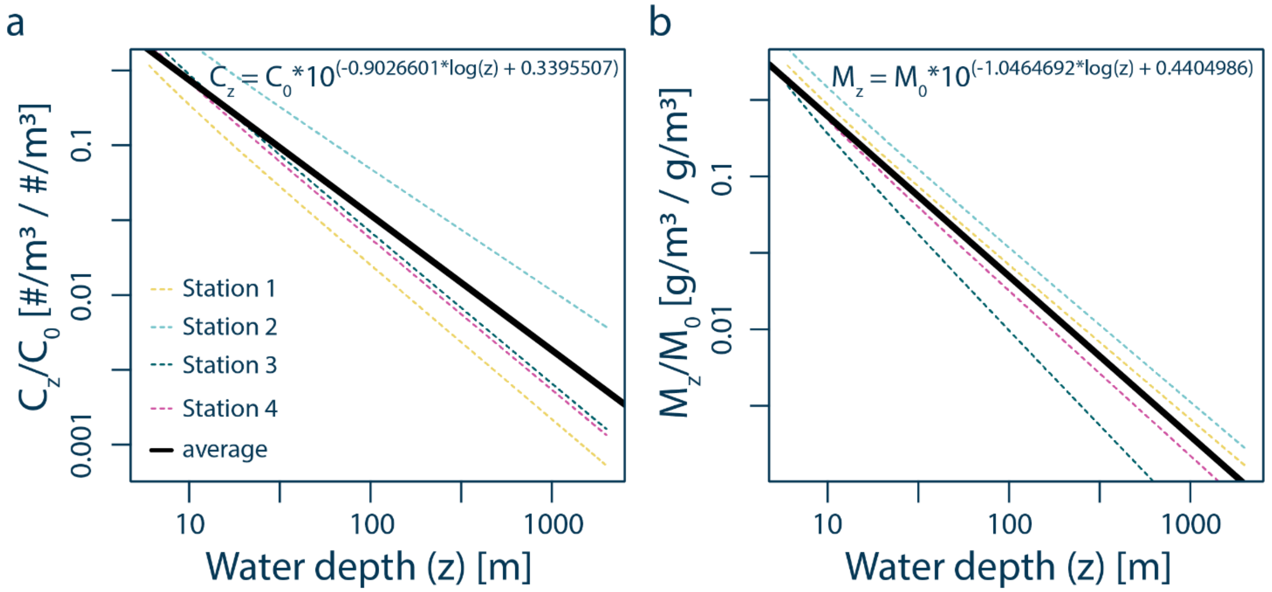

Fig. S5. Empirical relationships used to calculate the vertical transect plots (shown in Fig. 5). The modelled (a) numerical and (b) mass concentrations at water depth z (C_z and M_z) derived from the power law functions at Stations 1-4 are normalized to the corresponding concentrations in the upper 5 m of the water column (C_0 and M_0) and plotted versus water depth (z). The black solid line represents the average value for Stations 1-4, for which the equation is presented. This approach enables to estimate the concentration at a given water depth as a function of the surface concentrations.

Table S1. Station metadata and associated sampling schemes. Depth and volume correspond to the sampled water layer and water volume, respectively.

Sampling	Date	Sea State [Beaufort]	Coordinates Start	Coordinates End	Depth [m]	Volume [m^3]
			**Station 1**			
Manta 1_1	1/11/2018	3	25.140/-151.901	25.126/-151.900	0-0.15	212
MOCNESS 1_1	1/11/2018	3	25.160/-151.903	25.132/-151.901	1462-753	5039
MOCNESS 1_2	1/11/2018	3	25.132/-151.901	25.118/-151.899	753-436	1955
MOCNESS 1_3	1/11/2018	3	25.118/-151.899	25.102/-151.896	436-189	2420
MOCNESS 1_4	1/11/2018	3	25.102/-151.896	25.084/-151.888	189-107	2429
MOCNESS 1_5	1/11/2018	3	25.084/-151.888	25.065/-151.880	107-26	2709
MOCNESS 1_6	1/11/2018	3	25.065/-151.880	25.054/-151.876	26-6	381
MOCNESS 1_7	1/11/2018	3	25.054/-151.876	25.051/-151.875	6-2	136
			**Station 2**			
Manta 2_1	3/11/2018	3	28.663/-147.007	28.697/-147.010	0-0.15	388
Manta 2_2	3/11/2018	3	28.710/-147.015	28.732/-147.025	0-0.15	272
Manta 2_3	3/11/2018	3	28.740/-147.030	28.762/-147.037	0-0.15	402
MOCNESS 2_1	3/11/2018	3	28.716/-147.017	28.736/-147.027	1459-1188	3465
MOCNESS 2_2	3/11/2018	3	28.736/-147.027	28.752/-147.034	1188-599	2490
MOCNESS 2_3	3/11/2018	3	28.752/-147.034	28.770/-147.042	599-397	2528
MOCNESS 2_4	3/11/2018	3	28.770/-147.042	28.789/-147.049	397-195	2490
MOCNESS 2_5	3/11/2018	3	28.789/-147.049	28.807/-147.057	195-146	2515
MOCNESS 2_6	3/11/2018	3	28.807/-147.057	28.826/-147.066	146-73	2541
MOCNESS 2_7	3/11/2018	3	28.826/-147.066	28.842/-147.080	73-47	2426
MOCNESS 2_8	3/11/2018	3	28.842/-147.080	28.855/-147.097	47-4	2566
			**Station 3**			
Manta 3_1	13/11/2018	2	30.557/-145.043	30.575/-145.057	0-0.15	280
Manta 3_2	13/11/2018	2	30.581/-145.061	30.602/-145.077	0-0.15	293
Manta 3_3	13/11/2018	2	30.612/-145.083	30.633/-145.098	0-0.15	325
MOCNESS 3_1	13/11/2018	2	30.617/-145.086	30.687/-145.131	1868-412	4972
MOCNESS 3_2	13/11/2018	2	30.687/-145.131	30.704/-145.144	412-379	1485
MOCNESS 3_3	13/11/2018	2	30.704/-145.144	30.722/-145.156	379-193	2807
MOCNESS 3_4	13/11/2018	2	30.722/-145.156	30.741/-145.170	193-144	2677
MOCNESS 3_5	13/11/2018	2	30.741/-145.170	30.760/-145.184	144-66	2544
MOCNESS 3_6	13/11/2018	2	30.760/-145.184	30.780/-145.200	66-41	2073
MOCNESS 3_7	13/11/2018	2	30.780/-145.200	30.797/-145.214	41-21	1355
*MOCNESS 3_8**	*13/11/2018*	2	*30.797/-145.214*	*30.816/-145.231*	*21-10*	*2558*
MOCNESS 3a_1	15/11/2018	3	30.624/-145.090	30.655/-145.111	2001-1494	4850
MOCNESS 3a_2	15/11/2018	3	30.655/-145.111	30.672/-145.124	1494-1192	2671
MOCNESS 3a_3	15/11/2018	3	30.672/-145.124	30.698/-145.144	1192-595	4162
MOCNESS 3a_4	15/11/2018	3	30.698/-145.144	30.716/-145.157	595-394	2658
MOCNESS 3a_5	15/11/2018	3	30.716/-145.157	30.734/-145.171	394-193	2906
MOCNESS 3a_6	15/11/2018	3	30.734/-145.171	30.752/-145.184	193-144	2636
MOCNESS 3a_7	15/11/2018	3	30.752/-145.184	30.771/-145.198	144-62	2805
MOCNESS 3a_8	15/11/2018	3	30.771/-145.198	30.790/-145.214	62-2	2740
			**Station 4**			
Manta 4_1	1/12/2018	3	30.777/-133.435	30.780/-133.403	0-0.15	278
Manta 4_2	1/12/2018	3	30.780/-133.397	30.780/-133.375	0-0.15	186
Manta 4_3	1/12/2018	3	30.780/-133.365	30.778/-133.350	0-0.15	188
MOCNESS 4_1	1/12/2018	3	30.779/-133.358	30.776/-133.322	2002-1496	4521
MOCNESS 4_2	1/12/2018	3	30.776/-133.322	30.776/-133.302	1496-1193	2605
MOCNESS 4_3	1/12/2018	3	30.776/-133.302	30.780/-133.270	1193-496	4120
MOCNESS 4_4	1/12/2018	3	30.780/-133.270	30.782/-133.250	496-289	2310
MOCNESS 4_5	1/12/2018	3	30.782/-133.250	30.785/-133.227	289-188	2463
MOCNESS 4_6	1/12/2018	3	30.785/-133.227	30.787/-133.206	188-155	2348
MOCNESS 4_7	1/12/2018	3	30.787/-133.206	30.788/-133.185	155-55	2370
MOCNESS 4_8	1/12/2018	3	30.788/-133.185	30.790/-133.164	55-3	2285
			**Station 5**			
Manta 5_1	4/12/2018	5	31.548/-123.312	31.530/-123.312	0-0.15	272
Manta 5_2	4/12/2018	5	31.525/-123.310	31.507/-123.308	0-0.15	302
Manta 5_3	4/12/2018	5	31.503/-123.308	31.485/-123.305	0-0.15	282
MOCNESS 5_1	4/12/2018	5	31.516/-123.309	31.489/-123.307	1989-1487	4020
*MOCNESS 5_2**	*4/12/2018*	*5*	*31.489/-123.307*	*31.472/-123.303*	*1487-1194*	*2059*
MOCNESS 5_3	4/12/2018	5	31.472/-123.303	31.448/-123.294	1194-489	3789
MOCNESS 5_4	4/12/2018	5	31.448/-123.294	31.434/-123.285	489-290	2116
MOCNESS 5_5	4/12/2018	5	31.434/-123.285	31.419/-123.278	290-189	1874
MOCNESS 5_6	4/12/2018	5	31.419/-123.278	31.407/-123.267	189-142	1987
MOCNESS 5_7	4/12/2018	5	31.407/-123.267	31.396/-123.281	142-64	1911
MOCNESS 5_8	4/12/2018	5	31.396/-123.281	31.380/-123.294	64-3	2458

*Cod-end sample recovery failed.

Table S2. Power law functions to estimate water column plastic concentrations. The concentrations are calculated as a function of water depth: $C_{plastic} = 10^{(\mathbf{a}*\log(\text{Depth})+\mathbf{b})} * CF$. The correction factor (CF) is applied to account for the skewness bias inherent in the back conversion from a log-log transformed linear regression model to arithmetic units (high values lose significance relative to lower values), as described comprehensibly by [2], and was calculated using the variance of the model residuals [2]: $CF = e^{(2.65*\text{variance})}$.

[#/m^3]	**a**	**b**	**R^2**	**CF**
Station 1	-1.02748	-1.03254	0.9774	1.16
Station 2	-0.81053	-1.21204	0.9575	1.19
Station 3	-1.00905	-0.37732	0.9496	1.38
Station 4	-1.00631	-0.39438	0.9385	1.62
*Station 5**	*-0.39680*	*-2.49310*	*0.4082*	*2.16*
[g/m^3]	**a**	**b**	**R^2**	**CF**
Station 1	-1.0056	-3.8500	0.9078	1.86
Station 2	-1.0053	-3.5597	0.8980	2.00
Station 3	-1.2351	-2.9629	0.9122	2.41
Station 4	-1.0749	-3.5019	0.9410	1.70
*Station 5**	*-0.6244*	*-5.9980*	*0.6118*	*3.00*

*Note that Station 5 is excluded due to the absence of detectable plastic concentrations (i.e. $<10^{-4}$ #/m^3) in the deeper water column and the consequential large uncertainty in the regression model.

Table S3. Manta trawl numerical and mass concentrations results. ‘H-type’ plastics represent fragments and objects made of hard plastic, plastic sheet or film. ‘N-type’ stands for fragments of plastic lines, ropes, and fishing nets. ‘P-type’ are pre-production plastic pellets in the shape of a cylinder, disk or sphere. Note that no ‘F-type’ plastics (i.e. fragments or objects made of foamed material were found. All values were corrected for wind-induced mixing [3,4] as described in the method section.

	0.05-0.15cm		0.15-0.5cm			0.5-1.5cm		1.5-5cm		Total	
[#/km^2]	H	N	H	N	P	H	N	H	N	[#/km^2]	[#/m^3]
Manta 1_1	201195	-	57910	10731	4390	1483	19255	724	5080	300768	0.060
Manta 2_1	45003	-	75215	3910	1199	9321	1830	395	1586	138459	0.028
Manta 2_2	64895	-	88167	2784	1139	7502	2606	1689	4518	173299	0.035
Manta 2_3	20897	-	41175	-	-	4303	442	382	766	67965	0.014
Manta 3_1	214080	-	90559	5090	536	15004	16611	536	7502	349919	0.070
Manta 3_2	833001	-	398325	48034	5624	40905	35282	4602	7158	1372931	0.275
Manta 3_3	974493	-	345672	22926	5545	64235	31887	4621	15250	1464629	0.293
Manta 4_1	1396933	-	326082	5450	1115	15254	12118	1102	1659	1759712	0.352
Manta 4_2	299783	-	225072	8164	4175	5078	1911	-	1656	545839	0.109
Manta 4_3	1213486	-	431684	44384	-	10038	24552	-	7367	1731511	0.346
Manta 5_1	29015	-	15383	-	-	-	-	-	-	44398	0.009
Manta 5_2	-	-	-	-	-	-	2754	-	-	2754	0.0006
Manta 5_3	-	-	5945	-	-	-	-	-	-	5945	0.0012

	0.05-0.15cm		0.15-5cm			0.5-1.5cm		1.5-5cm		Total	
[kg/km^2]	H	N	H	N	P	H	N	H	N	[kg/km^2]	[µg/m^3]
Manta 1_1	0.0621	-	0.1898	0.0034	0.0565	0.0231	0.0128	0.1360	0.0065	0.490	98.0
Manta 2_1	0.0088	-	0.2772	0.0008	0.0058	0.4770	0.0013	0.1551	0.0266	0.953	190.5
Manta 2_2	0.0269	-	0.5142	0.0001	0.0184	0.2198	0.0014	0.3096	0.0054	1.096	219.2
Manta 2_3	0.0079	-	0.1575	-	-	0.1293	0.0002	0.0432	0.0046	0.343	68.6
Manta 3_1	0.1127	-	0.4072	0.0007	0.0115	0.3667	0.0104	0.0051	0.1188	1.033	206.6
Manta 3_2	0.4568	-	1.2074	0.0139	0.0470	0.9569	0.0369	4.1077	0.5334	7.360	1472.0
Manta 3_3	0.4741	-	1.7551	0.0047	0.0894	2.5424	0.0190	0.4053	0.0283	5.318	1063.7
Manta 4_1	0.8106	-	0.5462	0.0011	0.0060	0.2641	0.0187	0.0731	0.0028	1.723	344.5
Manta 4_2	0.0867	-	0.2989	0.0239	0.0057	0.0520	0.0013	-	0.0089	0.477	95.5
Manta 4_3	0.3656	-	0.4767	0.0143	-	0.1155	0.0258	-	0.0117	1.010	201.9
Manta 5_1	0.0053	-	0.0089	-	-	-	-	-	-	0.014	2.8
Manta 5_2	-	-	-	-	-	-	0.0011	-	-	0.001	0.2
Manta 5_3	-	-	-*	-	-	-	-	-	-	-	-

*Sample was lost during weighing.

Table S4. MOCNESS numerical concentrations results. 'H-type' plastics represent fragments and objects made of hard plastic, plastic sheet or film. 'N-type' stands for fragments of plastic lines, ropes, and fishing nets. Note that no 'P-type' (i.e. pre-production plastic pellets in the shape of a cylinder, disk or sphere) and 'F-type' plastics (i.e. fragments or objects made of foamed material) were found.

	0.05-0.15cm		0.15-0.5cm			0.5-1.5cm		1.5-5cm		5-10cm		10-50cm		Total
[#/m³]	H	N	H	N	P	H	N	H	N	H	N	H	N	[#/m³]
MOCNESS 1_1	-	-	-	-	-	-	-	-	-	-	-	-	-	-
MOCNESS 1_2	-	-	-	-	-	-	-	-	-	-	-	-	-	-
MOCNESS 1_3	-	-	0.0004	-	-	-	-	-	-	-	-	-	-	0.0004
MOCNESS 1_4	-	-	-	-	-	-	-	-	-	-	-	-	-	-
MOCNESS 1_5	-	-	-	-	-	-	-	-	-	-	-	-	-	-
MOCNESS 1_6	-	-	0.0026	-	-	-	-	-	-	-	-	-	-	0.0026
MOCNESS 1_7	0.0074	-	-	-	-	-	-	-	-	-	-	-	-	0.0074
MOCNESS 2_1	-	-	-	-	-	-	-	-	-	-	-	-	-	-
MOCNESS 2_2	-	-	-	-	-	-	-	-	-	-	-	-	-	-
MOCNESS 2_3	-	-	-	-	-	-	-	-	0.0004	-	-	-	-	0.0004
MOCNESS 2_4	-	-	0.0008	0.0004	-	-	0.0004	-	-	-	-	-	-	0.0016
MOCNESS 2_5	0.0008	-	0.0004	0.0004	-	-	-	-	-	-	-	-	-	0.0016
MOCNESS 2_6	0.0004	-	-	-	-	-	0.0008	-	-	-	-	-	-	0.0012
MOCNESS 2_7	0.0004	-	-	-	-	-	-	-	0.0004	-	-	-	-	0.0008
MOCNESS 2_8	0.0023	-	-	0.0004	-	-	0.0004	-	-	-	-	-	-	0.0031
MOCNESS 3_1	-	-	-	-	-	-	-	-	-	-	-	-	-	-
MOCNESS 3_2	0.0013	-	0.0007	0.0007	-	-	-	-	-	-	-	-	-	0.0027
MOCNESS 3_3	0.0007	-	0.0004	0.0004	-	-	-	-	-	-	-	-	-	0.0014
MOCNESS 3_4	-	-	-	-	-	-	-	-	-	-	-	-	-	-
MOCNESS 3_5	0.0004	-	0.0004	-	-	0.0008	-	-	-	-	-	-	-	0.0016
MOCNESS 3_6	-	-	-	-	-	-	-	-	-	-	-	-	-	-
MOCNESS 3_7	0.0221	-	0.0022	0.0022	-	-	0.0037	-	0.0015	-	0.0007	-	-	0.0325
MOCNESS 3a_1	-	-	-	-	-	-	-	-	-	-	-	-	-	-
MOCNESS 3a_2	-	-	0.0004	-	-	-	-	-	0.0004	-	-	-	-	0.0007
MOCNESS 3a_3	-	-	-	-	-	-	-	-	-	-	-	-	-	-
MOCNESS 3a_4	-	-	-	0.0004	-	-	0.0008	-	-	-	-	-	-	0.0011
MOCNESS 3a_5	-	-	-	-	-	-	0.0003	-	-	-	-	-	-	0.0003
MOCNESS 3a_6	0.0011	-	-	-	-	-	0.0004	-	-	-	-	-	-	0.0015
MOCNESS 3a_7	0.0011	-	0.0004	-	-	-	0.0004	-	-	-	-	-	-	0.0018
MOCNESS 3a_8	0.0088	-	0.0029	0.0015	-	0.0004	0.0026	-	0.0011	-	-	-	-	0.0172
MOCNESS 4_1	0.0002	-	-	-	-	-	0.0007	-	-	-	-	-	-	0.0009
MOCNESS 4_2	-	-	-	-	-	-	-	-	-	-	-	-	-	-
MOCNESS 4_3	0.0005	-	0.00049	-	-	-	-	-	0.0002	-	-	-	-	0.0012
MOCNESS 4_4	0.0004	-	-	0.0004	-	-	0.0009	-	-	-	-	-	-	0.0017
MOCNESS 4_5	-	-	-	0.0004	-	-	-	-	-	-	-	-	-	0.0004
MOCNESS 4_6	-	-	0.0004	-	-	-	-	-	-	-	-	-	-	0.0004
MOCNESS 4_7	0.0013	-	0.0008	-	-	-	-	-	-	-	-	-	-	0.0021
MOCNESS 4_8	0.0044	-	0.0004	0.0009	-	-	0.0022	-	0.0004	-	-	-	-	0.0083
MOCNESS 5_1	-	-	-	-	-	-	-	-	-	-	-	-	-	-
MOCNESS 5_3	-	-	-	-	-	-	-	-	-	-	-	-	-	-
MOCNESS 5_4	-	-	-	-	-	-	-	-	-	-	-	-	-	-
MOCNESS 5_5	-	-	-	-	-	-	-	-	-	-	-	-	-	-
MOCNESS 5_6	-	-	-	-	-	-	-	-	-	-	-	-	-	-
MOCNESS 5_7	-	-	-	-	-	-	-	-	-	-	-	-	-	-
MOCNESS 5_8	0.0012	-	-	-	-	-	-	-	-	-	-	-	-	0.0012

"-": Values below detection limit (~10^{-4} #/m³, depending on filtered water volume, see Supplementary Table S1)

Table S5. MOCNESS mass concentrations results. ‘H-type’ plastics represent fragments and objects made of hard plastic, plastic sheet or film. ‘N-type’ stands for fragments of plastic lines, ropes, and fishing nets. Note that no ‘P-type’ (i.e. pre-production plastic pellets in the shape of a cylinder, disk or sphere) and ‘F-type’ plastics (i.e. fragments or objects made of foamed material) were found.

	**0.05-0.15cm**		**0.15-0.5cm**			**0.5-1.5cm**		**1.5-5cm**		**5-10cm**		**10-50cm**		**Total**
[μg/m³]	H	N	H	N	P	H	N	H	N	H	N	H	N	[μg/m³]
MOCNESS 1_1	-	-	-	-	-	-	-	-	-	-	-	-	-	-
MOCNESS 1_2	-	-	-	-	-	-	-	-	-	-	-	-	-	-
MOCNESS 1_3	-	-	0.91	-	-	-	-	-	-	-	-	-	-	0.91
MOCNESS 1_4	-	-	-	-	-	-	-	-	-	-	-	-	-	-
MOCNESS 1_5	-	-	-	-	-	-	-	-	-	-	-	-	-	-
MOCNESS 1_6	-	-	7.35	-	-	-	-	-	-	-	-	-	-	7.35
MOCNESS 1_7	3.68	-	-	-	-	-	-	-	-	-	-	-	-	3.68
MOCNESS 2_1	-	-	-	-	-	-	-	-	-	-	-	-	-	-
MOCNESS 2_2	-	-	-	-	-	-	-	-	-	-	-	-	-	-
MOCNESS 2_3	-	-	-	-	-	-	-	-	0.47	-	-	-	-	0.47
MOCNESS 2_4	-	-	9.32	0.24	-	-	0.28	-	-	-	-	-	-	9.84
MOCNESS 2_5	0.08	-	2.07	-	-	-	-	-	-	-	-	-	-	2.15
MOCNESS 2_6	1.22	-	-	-	-	-	0.35	-	-	-	-	-	-	1.57
MOCNESS 2_7	0.54	-	-	-	-	-	-	-	0.49	-	-	-	-	1.03
MOCNESS 2_8	1.17	-	-	0.23	-	-	0.19	-	-	-	-	-	-	1.60
MOCNESS 3_1	-	-	-	-	-	-	-	-	-	-	-	-	-	-
MOCNESS 3_2	2.15	-	1.41	0.20	-	-	-	-	-	-	-	-	-	3.77
MOCNESS 3_3	0.04	-	0.21	0.04	-	-	-	-	-	-	-	-	-	0.29
MOCNESS 3_4	-	-	-	-	-	-	-	-	-	-	-	-	-	-
MOCNESS 3_5	0.08	-	0.28	-	-	1.45	-	-	-	-	-	-	-	1.81
MOCNESS 3_6	-	-	-	-	-	-	-	-	-	-	-	-	-	-
MOCNESS 3_7	6.27	-	7.68	0.44	-	-	2.07	-	3.47	-	3.10	-	-	23.03
MOCNESS 3a_1	-	-	-	-	-	-	-	-	-	-	-	-	-	-
MOCNESS 3a_2	-	-	2.13	-	-	-	-	-	0.15	-	-	-	-	2.28
MOCNESS 3a_3	-	-	-	-	-	-	-	-	-	-	-	-	-	-
MOCNESS 3a_4	-	-	-	0.04	-	-	0.26	-	-	-	-	-	-	0.30
MOCNESS 3a_5	-	-	-	-	-	-	0.21	-	-	-	-	-	-	0.21
MOCNESS 3a_6	0.23	-	-	-	-	-	0.11	-	-	-	-	-	-	0.34
MOCNESS 3a_7	1.03	-	0.61	-	-	-	0.29	-	-	-	-	-	-	1.93
MOCNESS 3a_8	4.89	-	12.81	0.11	-	2.04	1.31	-	2.74	-	-	-	-	23.91
MOCNESS 4_1	0.02	-	-	-	-	-	0.31	-	-	-	-	-	-	0.33
MOCNESS 4_2	-	-	-	-	-	-	-	-	-	-	-	-	-	-
MOCNESS 4_3	0.10	-	0.66	-	-	-	-	-	0.51	-	-	-	-	1.26
MOCNESS 4_4	0.04	-	-	-	-	-	0.22	-	-	-	-	-	-	0.26
MOCNESS 4_5	-	-	-	0.16	-	-	-	-	-	-	-	-	-	0.16
MOCNESS 4_6	-	-	0.43	-	-	-	-	-	-	-	-	-	-	0.43
MOCNESS 4_7	0.34	-	1.22	-	-	-	-	-	-	-	-	-	-	1.56
MOCNESS 4_8	1.93	-	1.66	0.18	-	-	1.05	-	1.01	-	-	-	-	5.82
MOCNESS 5_1	-	-	-	-	-	-	-	-	-	-	-	-	-	-
MOCNESS 5_3	-	-	-	-	-	-	-	-	-	-	-	-	-	-
MOCNESS 5_4	-	-	-	-	-	-	-	-	-	-	-	-	-	-
MOCNESS 5_5	-	-	-	-	-	-	-	-	-	-	-	-	-	-
MOCNESS 5_6	-	-	-	-	-	-	-	-	-	-	-	-	-	-
MOCNESS 5_7	-	-	-	-	-	-	-	-	-	-	-	-	-	-
MOCNESS 5_8	0.08	-	-	-	-	-	-	-	-	-	-	-	-	0.08

Table S6. Areal depth integrated concentrations for plastic debris (500 µm to 5 cm in size) in the 0-5 m and 5-2000 m water layer (diamonds shown in Fig. 4). Values for the 0-5 m water layer represent measured concentrations of floating plastic debris at the ocean surface collected by Manta trawls and corrected for wind-induced mixing in the upper 5 m of water column. Values for the 5-2000 m water layer are derived by integrating the corresponding power law functions (Fig. 2; Supplementary Table S2) using integration steps of 1 m. Note that no power law function is available for Station 5.

	0-5 m		5-2000 m	
	[#/km^2]	[kg/km^2]	[#/km^2]	[kg/km^2]
Station 1	0.30e6	0.490	0.57e6	1.537
Station 2	0.13e6	0.797	1.11e6	3.232
Station 3	1.06e6	4.571	3.33e6	5.782
Station 4	1.35e6	1.070	3.81e6	2.289

SUPPLEMENTARY REFERENCES

1. Hansell, D. A., Carlson, C. A. & Suzuki, Y. Dissolved organic carbon export with North Pacific Intermediate Water formation. *Global Biogeochem. Cycles* **16**, 1–8 (2002).
2. Middelburg, J. J., Soetart, K. & Herman, P. M. J. Empirical relationships for use in global diagenetic models. *Deep. Res. I* **44**, 327–344 (1997).
3. Kukulka, T., Proskurowski, G., Morét-Ferguson, S., Meyer, D. W. & Law, K. L. The effect of wind mixing on the vertical distribution of buoyant plastic debris. *Geophys. Res. Lett.* **39**, 1–6 (2012).
4. Lebreton, L. *et al.* Evidence that the Great Pacific Garbage Patch is rapidly accumulating plastic. *Sci. Rep.* **8**, 1–15 (2018).
